# Supplementary material for: Splice-Junction-Based Mapping of Alternative Isoforms in the Human Proteome
Source: Cell Rep. Author manuscript; Available in PMC 2020 Jan 15. (PMC6961840; doi:10.1016/j.celrep.2019.11.026)
Supplement: 2 [file NIHMS1546469-supplement-2.zip › DF1/PXD009737/TestisGluC/TestisGluC_10_SORBS1_SYLLQPSYPAKDATSSSAAQPE.pdf]

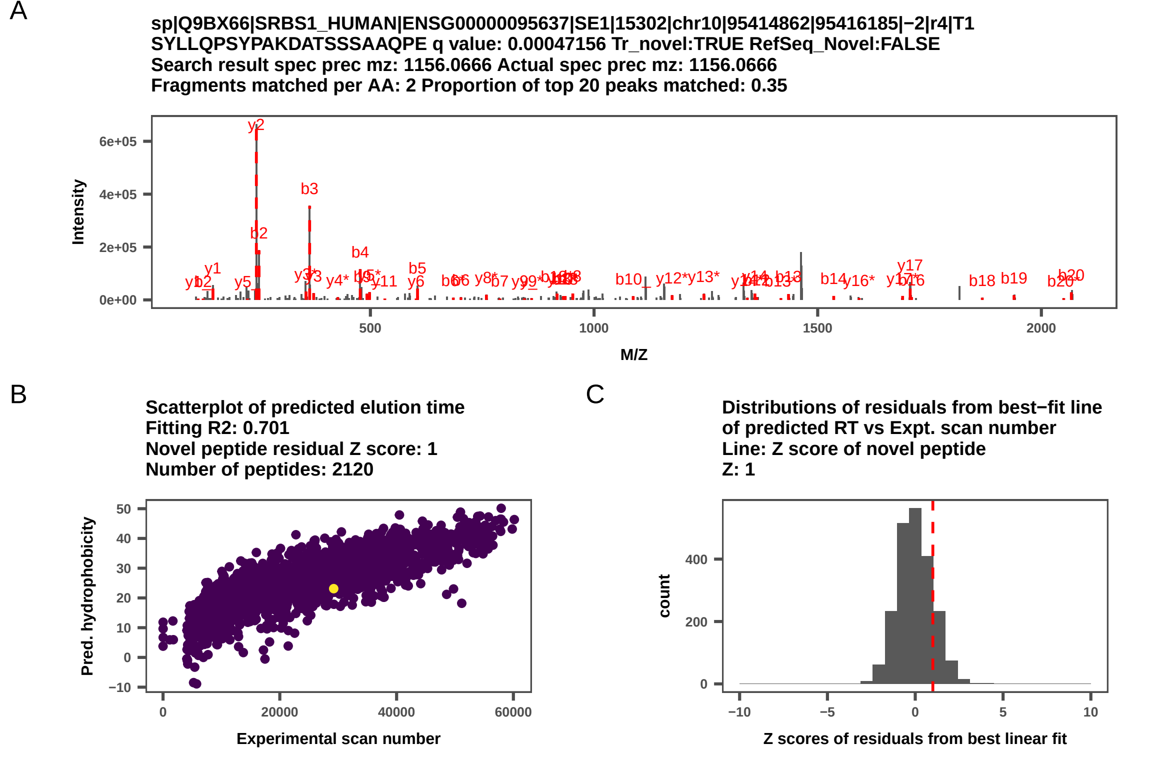
A
sp|Q9BX66|SRBS1_HUMAN|ENSG00000095637|SE1|15302|chr10|95414862|95416185|−2|r4|T1
SYLLQPSYPAKDATSSSAAQPE q value: 0.00047156 Tr_novel:TRUE RefSeq_Novel:FALSE
Search result spec prec mz: 1156.0666 Actual spec prec mz: 1156.0666
Fragments matched per AA: 2 Proportion of top 20 peaks matched: 0.35
Intensity
6e+05
4e+05
2e+05
0e+00
500
1000
1500
2000
M/Z
y2
b3
b2
b4
b5
y1
y5
y6
b10
b14
y17
b18
b19
b20
B
Scatterplot of predicted elution time
Fitting R2: 0.701
Novel peptide residual Z score: 1
Number of peptides: 2120
Pred. hydrophobicity
50
40
30
20
10
0
−10
0
20000
40000
60000
Experimental scan number
C
Distributions of residuals from best−fit line
of predicted RT vs Expt. scan number
Line: Z score of novel peptide
Z: 1
count
400
200
0
−10
−5
0
5
10
Z scores of residuals from best linear fit
